# Topoisomerase IIIα resolves inter- and intra-molecular intertwines during DNA replication

**DOI:** 10.21203/rs.3.rs-11133141/v1

**Published:** 2026-10-19

**Authors:** James Dewar, Sabrina Van Ravenstein, Lillian Campos, Gabor Harami, Darren Heintzman, Steven Dahmen, William Dunphy, Keir Neuman

## Abstract

Resolution of topological stress is crucial for genome integrity. Vertebrate topoisomerase IIα (TOP2α) resolves catenanes to relieve topological stress and unlink daughter molecules during DNA replication. Topoisomerase IIIα (TOP3α) can also resolve catenanes, but its direct role during DNA replication, substrate specificity, and relevant binding partners remain unclear. Here we show that TOP3α directly resolves replication-generated DNA intertwines in Xenopus egg extracts. When TOP2α function is compromised, TOP3α promotes replication fork progression and daughter strand unlinking preferentially during replication termination. TOP3α can carry out this role independently of its binding partners RMI1-RMI2 and the BLM helicase by acting on lagging-strand single-stranded DNA (ssDNA). Strikingly, elevated lagging-strand ssDNA drives formation of intramolecular ssDNA intertwines even when TOP2α is present, and TOP3α resolves these structures. Thus, lagging-strand ssDNA provides a substrate for TOP3α to promote replication termination but can also generate intramolecular intertwines that require TOP3α for their resolution.

